# Development of a rapid and reliable surveillance method for *Ornithodoros turicata americanus* in gopher tortoise (*Gopherus polyphemus*) burrows in the southeastern United States

**DOI:** 10.1111/mve.12764

**Published:** 2024-09-11

**Authors:** Nicholas Canino, Carson Torhorst, Sebastian Botero‐Cañola, Lorenza Beati, Kathleen C. O'Hara, Angela James, Samantha M. Wisely

**Affiliations:** ^1^ Department of Wildlife Ecology and Conservation University of Florida Gainesville Florida USA; ^2^ US National Tick Collection, Institute for Coastal Plain Science, Georgia Southern University Statesboro Georgia USA; ^3^ Center for Epidemiology and Animal Health, Veterinary Services, Animal and Plant Health Inspection Service, USDA Fort Collins Colorado USA

**Keywords:** African swine fever virus, burrow vacuuming, carbon dioxide traps, detection probability, nidicolous, occupancy model, sample extraction

## Abstract

The soft tick *Ornithodoros turicata* Duges (Acari: Argasidae) is a potential vector of African swine fever virus (ASFV). We evaluated the efficacy of two methods to collect soft ticks rapidly and efficiently from gopher tortoise (*Gopherus polyphemus*) burrows, which are ubiquitous throughout large regions of the southeastern United States and their burrows are a known microhabitat of *O. turicata*. Burrow vacuuming was an effective and efficient tick collection method; no tick was captured employing CO_2_ trapping. Using an occupancy modelling framework, we estimated that the probability of detecting ticks from an infested burrow each time a sample was taken with this method was 58% and increased with the average relative humidity. With the occupancy model, we estimated that 70% of the burrows in the study area were infested with *O. turicata*. Manual sifting of the burrow material yielded more ticks (6.6 individuals/sample) than using a set of three sieves (2.9 individuals/sample), yet the probability of detecting the species was not different between the two methods (Pval = 0.7). These methods can inform the development of ASF vector surveillance and outbreak response plans in areas of high risk for ASFV introduction in the region.

## Graphical abstract

- We developed a rapid, effective technique for surveying for *Ornithodoros turicata*, a vector of African swine fever virus using gopher tortoise burrows.
- When working under a time constraint during tick collection, vacuuming substrate from the top meter of the burrow followed by sifting for soft ticks outperformed CO_2_ traps.
- Probability of detection of soft ticks in gopher tortoise burrows decreased as relative humidity decreased.

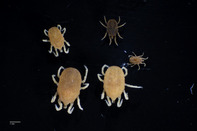

## INTRODUCTION

Soft ticks belonging to the genus *Ornithodoros* (Acari: Argasidae) serve as vectors for various pathogens that impact both humans and animals globally. In Africa and Europe, *Ornithodoros* ticks are involved in the transmission of African swine fever virus (ASFV), a pathogen that impacts wild and domestic pigs (Galindo & Alonso, 2017). In North America, the species *Ornithodoros turicata americanus* collected from peninsular Florida was a competent vector of this virus in laboratory experiments (Golnar et al., 2019; Hess et al., 1987). Although ASFV has not been reported in the United States (US), certain regions, including the southeastern United States, have been identified as high risk for introduction of the virus (USDA, 2023). The World Organisation for Animal Health (WOAH) Terrestrial Code recommends *Ornithodoros* tick surveillance to demonstrate no evidence of the presence or involvement of this vector when declaring ASF disease freedom, yet very little is known about the geographic distribution of *O. turicata* in the southeastern United States. As a first step to developing a surveillance strategy, effective and efficient methodologies to collect and evaluate soft tick vector samples during an outbreak are critical to the response effort (Pfeiffer et al., 2021).

ASF is responsible for a global panzootic in domestic and wild pigs, resulting in substantial economic losses globally (Dixon et al., 2020; Gaudreault et al., 2020; Mason‐D'Croz et al., 2020; Nguyen‐Thi et al., 2021). In 2021, ASFV was detected in the Caribbean, the first record in the western hemisphere in nearly four decades (Ruiz‐Saenz et al., 2022). This discovery raises concerns about the potential spread of the virus to the United States, increasing the global reach of this pathogen. The state of Florida is an important entry point to North America for invasive vector‐borne pathogens including Zika Virus, Dengue Virus and Chikungunya Virus (Alto et al., 2017; Teets et al., 2014; Zimler & Alto, 2019). Florida maintains a high volume of commerce and travel around the Caribbean, which increases the risk of human transportation of the virus into the region (USDA, 2023). In addition, the southeastern United States boasts high population densities of invasive wild pigs (*Sus scrofa*), maintains a substantial commercial pork industry and contains a subspecies of the potential vector, *O. turicata americanus* (Beck et al., 1986; Lewis et al., 2019). The convergence of these factors creates the conditions for a naturalised sylvatic cycle and expansion of ASFV into important regions for the American pork industry. These factors underscore the necessity for the development of a standardised protocol for field collection of these vectors that is rapid, efficient and easy to deploy.

*Ornithodoros turicata* is a nidicolous tick; they live in nests, burrows and crevices and spend limited time attached to their host. These ticks have a generalist diet and are known to feed on a wide variety of hosts, ranging from reptiles and birds to mammals, including domestic dogs and humans (Cooley & Kohls, 1944). There are several records of *O. turicata* feeding on pigs (Cooley & Kohls, 1944; Sames & Teel, 2022), but there have been no studies describing the host relationship with invasive wild pigs in the United States, a crucial factor in understanding the potential role of this tick species in the transmission of ASFV. Habitat associations of *O. turicata americanus* in the southeastern United States are not well understood; most records of this species in Florida were collected from gopher tortoise (*Gopherus polyphemus*) burrows. These burrows serve as critical microhabitats for soft ticks, where collections of >1000 individuals per burrow have occurred (Adeyeye & Butler, 1989). Nonetheless, little is known about the large‐scale habitat association or the geographic and climatic conditions that influence the distribution and magnitude of Argasid tick infestation in gopher tortoise burrows.

Several methodologies have been deployed to collect Argasid ticks from vertebrate burrows and natural crevices in the field. Manual excavation of burrows and then screening their contents, a time‐consuming and destructive method, has been largely replaced by CO_2_ trapping and burrow vacuuming (Butler & Holscher, 1984; Milstrey, 1987). CO_2_ trapping has been shown to attract ticks from up to 8 m from a burrow entrance and allows an easy way to mark individuals for population studies (Adeyeye & Butler, 1991). Burrow vacuuming allows researchers to collect large numbers of ticks and define microhabitats within the burrow (Adeyeye & Butler, 1989; Butler & Holscher, 1984). In addition to methodological approaches, detectability of *O. turicata* can also be influenced by environmental variables such as relative humidity and rainfall (Adeyeye & Butler, 1989), yet the efficacy of these methods for detecting and collecting *Ornithodoros* species under diverse conditions is not well understood.

Currently, there is no standardised methodology for field surveillance of Argasid tick populations. Given the pressing need for an efficient and easily deployable field collection method for *O. turicata*, we evaluated several survey methodologies for detecting soft ticks in gopher tortoise burrows in north central Florida with the aim of informing future survey designs that would be applicable to the southeastern United States. We chose to confine our surveys to gopher tortoise burrows because of their ubiquitous presence in multiple ecoregions of the southeastern United States, their high visibility within those ecoregions, and their known association with *O. t. americanus*. Although confining surveys to this microhabitat would not increase our understanding of the diversity of microniches used by this subspecies, it has the potential to provide a rapid and reliable means of collecting Argasid ticks across a large region of the southeastern United States and may thus be an efficacious survey technique to include in an ASFV response plan.

The objective of our research was to establish a rapid and repeatable surveillance method for *O. turicata* in gopher tortoise burrows. In this study, we sampled Argasid ticks in a north central Florida Gulf Coast Forest ecosystem using two distinct methods with the following aims: (i) to assess the probability of detection and the number of ticks collected using a time limited CO_2_ trap versus a spatially limited vacuum technique; (ii) to estimate burrow occupancy in the landscape and evaluate the influence of local and landscape variables on the probability of detection; and (iii) to evaluate the efficiency and reliability of two different techniques for extracting ticks from substrate samples collected in gopher tortoise burrows.

## MATERIALS AND METHODS

### Ethics and safety

Prior to conducting field work, permits were obtained from the Florida Fish and Wildlife Conservation Commission to sample around and within gopher tortoise burrows (Permit N. LSSC‐22‐00054). These burrows are frequently used by various species, including the threatened gopher frog (*Lithobates capito*), the vulnerable Florida mouse (*Podomys floridanus*) and the federally threatened eastern indigo snake (*Drymarchon couperi*). As a result, our protocol included assessing the presence of any vertebrate within each burrow prior to sampling. A borescope (Teslong NTS300) was used to a depth of 2 m within each burrow to assess the presence of vertebrates. Burrows were not sampled if any vertebrates were detected. Additionally, to minimise the disturbance of gopher tortoises, a threatened species in Florida (Enge et al., 2006), the research permit required the CO_2_ traps to be deployed for no more than 30 min, and at 1 m of the burrow entrance, and vacuuming to take place at a maximum depth of 1 m. Finally, all equipment that entered the burrow was disinfected using an antimicrobial solution of 0.2% Chlorohexidine acetate (Nolvasan) to avoid pathogen spread between sampled burrows. Because this work did not involve manipulating any vertebrate, an Institutional Animal Care and Use (IACUC) protocol at the University of Florida was not required.

### Study area

**FIGURE 1:**
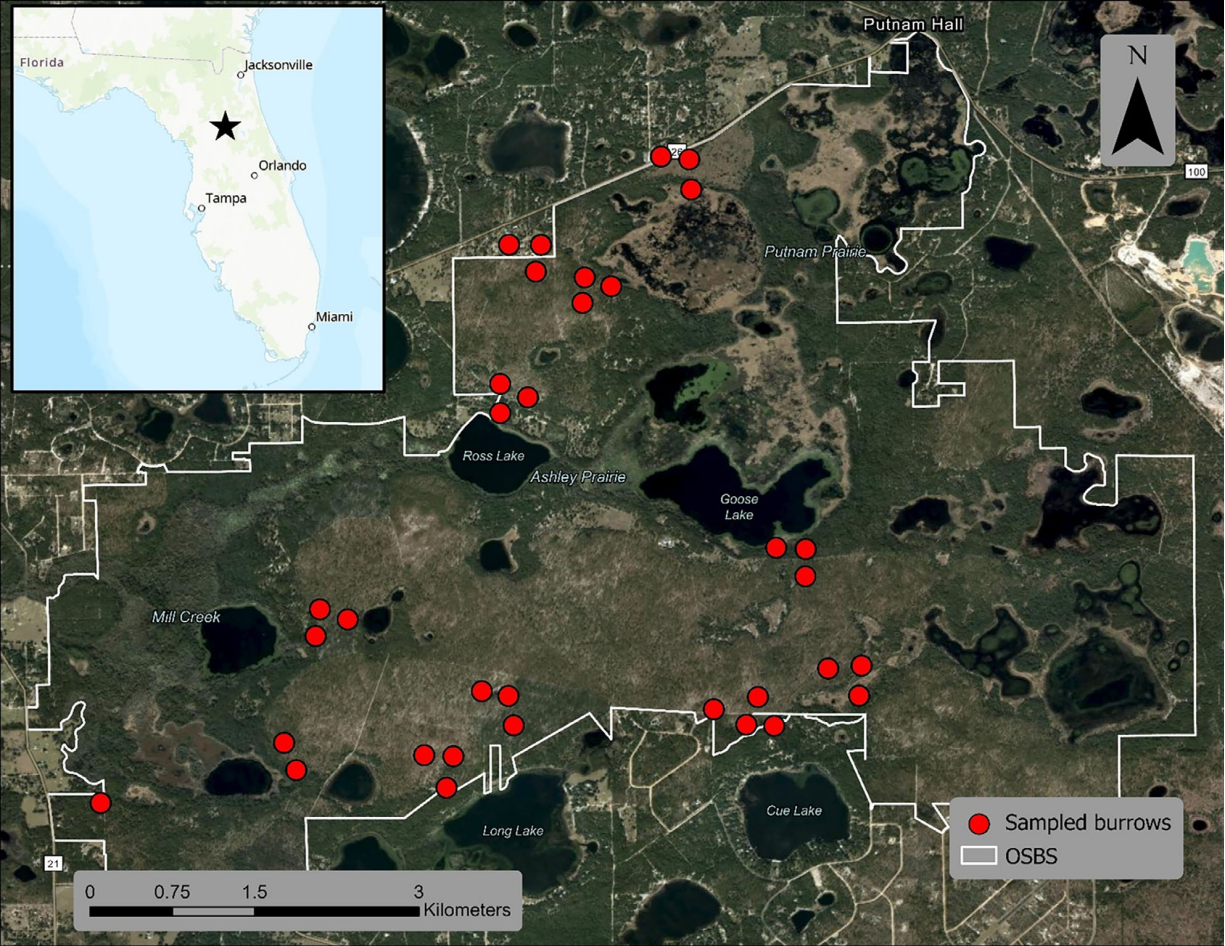
Sampling of gopher tortoise burrows for *Ornithodoros turicata* study area. Limits of Ordway‐Swisher Biological Station, Florida, and the geographic distribution of sampled burrows.

The field portion of this work took place between October 2022 and January 2023 at Ordway‐Swisher Biological Station (OSBS) in north central Florida. The biological station is part of the Florida Ridge ecoregion, and the land cover consists primarily of high pine forests with interspersed hammock and wetland communities (Myers & Ewel, 1990; Omernik, 1987; Figure 1).

### Field collection methods

Our study assessed the efficacy of two field methods previously used for collecting soft ticks: the use of CO_2_ traps and vacuuming burrow material (Adeyeye & Butler, 1989; Butler & Holscher, 1984; Milstrey, 1987). Burrows were selected based on accessibility from roads and with the aim of covering diverse habitats within the study area (Figure 1). At the time of sample collection, we measured relative humidity and air temperature at the entrance of the burrow using a thermohygrometer (Kestrel 5000 Environmental Meter or Digi‐Sense 2025011). We also recorded the burrow dimensions (height and width), coordinates and time of sampling event. Finally, burrow activity level was assessed for a subset of the burrows.

**FIGURE 2:**
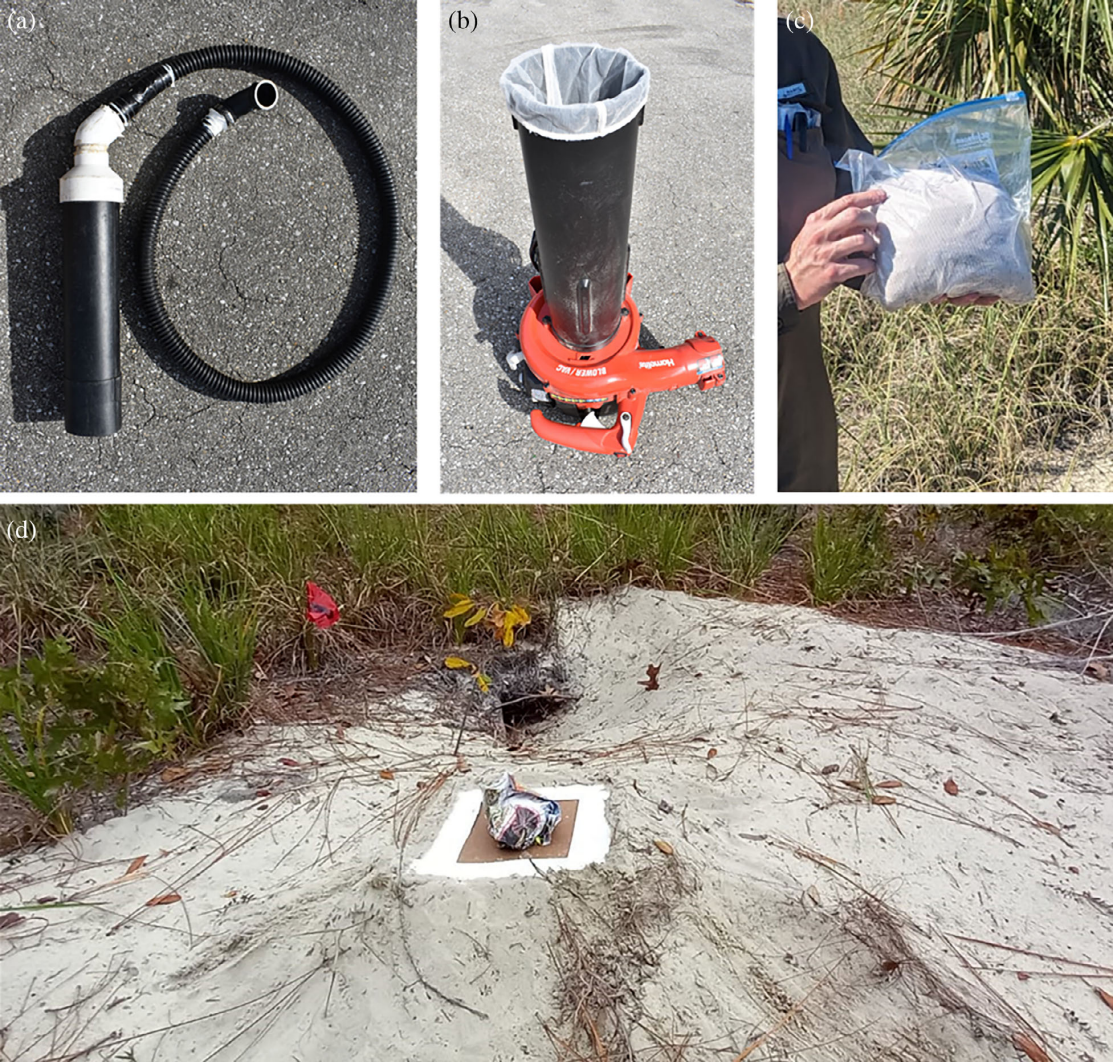
Gopher tortoise burrow sampling for *Ornithodoros turicata*: field collection methods and equipment. (a) A 4‐cm internal diameter hose attached to the lower vacuum tube through a PVC elbow and adapter. The posterior end of the hose includes a rubber elbow to facilitate sample collection. (b) A Homelite 26 cc gas‐powered blower/vacuum with collection mesh installed on the upper vacuum tube. (c) One 3.8‐L resealable polyethylene bag containing the mesh with approximately 2000 g of the collected burrow material. (d) CO_2_ trap installed 1 m from a burrow entrance and consisting of a 30 × 30 cm pressed‐wood surface, on which 450 g of dry ice, covered by porous paper were placed at the centre and surrounded by glue traps (10 cm × 25 cm Terro insect traps).

At 23 burrows, CO_2_ traps were installed 1 m from the entrance of the burrow with each side level with the soil (Figure 2d). The CO_2_ trap consisted of a 50 × 50 or 30 × 30 cm pressed‐wood surface, on which approximately 450 g of dry ice were placed at the centre and surrounded by glue traps (10 cm × 25 cm Terro insect traps) (Busselman et al., 2021). Traps were removed after 30 min, and then we used the vacuum method at the same burrow.

At 36 burrows we used a modified version of the vacuum system described by Butler et al. (Butler & Holscher, 1984) and Milstrey (1987). This system consisted of a Homelite® 26 cc gas‐powered blower/vacuum (4.25 to 11.3 m^3^/min) with a 4‐cm internal diameter hose attached to the lower vacuum tube through a polyvinyl chloride (PVC) pipe elbow and adapter (Figure 2a). The hose had a length of 2–4 m with a rubber elbow attached to the end opposite to the vacuum and a 1‐m wood dowel fastened to this end to facilitate vacuuming within the burrow. A High Flow Mesh Filter Media Bag (Aquatic Bags for Filter Media—8 × 12 inch and 100 μm high flow mesh) was installed between the lower and upper vacuum tubes to collect the extracted sample (Figure 2b). We aimed to collect enough burrow material to completely fill the 3.8‐L mesh bag. Loose substrate and debris were vacuumed from the top, bottom and sides down to 1 m of each gopher tortoise burrow. The mesh bags containing the sample were placed in a 3.8‐L resealable polyethylene bag (Ziploc®, S. C. Johnson & Son, Racine, WI) and labelled for storage before processing (Figure 2c).

We sampled 23 gopher tortoise burrows using both the CO_2_ method immediately followed by the vacuum method. After sampling 23 burrows, we abandoned the CO_2_ method because we did not collect any soft ticks with this method, even though we did collect soft ticks using the vacuum method (with or without CO_2_ beforehand). We sampled an additional 13 gopher tortoise burrows using only the burrow vacuum method (Milstrey, 1987). To assess the probability of detecting *O. turicata* in infested burrows, each burrow was sampled repeatedly up to three times, with a minimum interval of 1 week between sampling events.

### Sample examination

From each sampling event using the burrow vacuum, we obtained a 3.8‐L polyethylene plastic bag containing the mesh bag with burrow substrate material (mean weight = 1750 g) from which ticks were collected. We tested two methods to collect the soft ticks from the substrate, both of which took advantage of the fact that the legs of *O. turicata* fluoresce blue‐green when exposed to UV light (Butler & Holscher, 1984). All polyethylene bags were weighed before being processed by either method. The first examination method, manual sifting, consisted of pouring the material from the bags through a 1.5‐mm handheld strainer. The debris in the strainer was then carefully examined under an ultraviolet (UV) light (Glossday Blacklight Flashlight, 18 Watts, 128 LED UV—395‐nm wavelength) for soft ticks. Afterwards, the substrate that passed through the strainer was evenly distributed on a white plastic tray using a fine paint brush. Once all the sand was thinly spaced out, the UV light was once again used to examine the tray for soft ticks. The use of a white tray allowed for the fluorescence of the ticks to reflect more clearly and made the ticks easier to locate.

The second examination method, sieving, was a system that used three screens of decreasing mesh sizes (1.6, 1.2 and 1.0 mm). Before sieving, the polyethylene and mesh bag were visually examined for ticks. Afterwards, the sample was sieved through the 1.6‐mm mesh screen into a plastic container. The debris trapped in the sieve was thoroughly examined directly on the screen, where active ticks were easily spotted. Then, the debris was transferred to a tray to detect hidden and inactive ticks that could only be spotted by their fluorescence. The same process was repeated for the following two screens with the material that passed through the previous screen. The substrate that passed through the last screen was not examined for soft ticks. For polyethylene bags with substrate that was too damp to pass through the sieve, we poured the material into a plastic container for 24 to 48 h to dry before examination. We placed double‐sided tape along the rim of the container to prevent ticks escaping and removed the trapped ticks before sieving.

The recovered soft ticks were identified to stage and to sex for adults. All soft ticks collected were also identified to species via examination of diagnostic characteristics under a dissecting microscope. Collected individuals were compared to reference specimens from the United States National Tick Collection. We identified *O. turicata* from other species potentially occurring in the region such as *Ornithodoros talaje, Ornithodoros parkeri* and *Ornithodoros nicollei* using the following characters: absent cheeks, dorsoventral grooves present, dorsal humps on tarsi I present, absent eyes, subapical dorsal protuberance on leg IV absent and the presence of large mammillae less crowded that in specimens of *O. parkeri* (Cooley & Kohls, 1944). After identification, all soft ticks were stored in 1.5‐mL microcentrifuge tubes with 95% molecular grade ethanol for downstream molecular analyses of bloodmeal identity and pathogen surveillance.

### Statistical analysis

To explore the environmental factors influencing the detectability and occurrence of *O. turicata* among burrows at OSBS, we analysed the tick detection data collected using the burrow vacuuming method in an occupancy framework. This approach is based on the creation of a hierarchical model where two parameters are simultaneously estimated: the probability of occupancy (*ψ*; percentage of infested burrows) and the probability of detection (*p*; probability of detecting the species given that the burrow is infested). Occupancy models not only provide more robust estimates of *ψ* and *p*, but also allow the researcher to model these parameters as functions of covariates, enhancing our understanding of the factors influencing these components (MacKenzie et al., 2002). We created a single season occupancy model of *O. turicata* at OSBS using the detection data from 36 burrows vacuumed on at least one and up to three sampling occasions.

We explored the influence of three variables on the probability of a burrow being infested (Table 1): (i) Topographic Wetness Index (TWI), a measure of the capacity of a location to retain water based on the surrounding topography and calculated using a digital elevation model (Besnard et al., 2013). We hypothesized burrow occupancy would be negatively influenced by TWI as tick activity and survival decrease under damp conditions (Adeyeye & Butler, 1989); (ii) time since last fire (Fire), which is one of the main disturbances occurring in the study area, and could have negative indirect effects on tick occurrence through changes in host communities and microclimate (Gallagher et al., 2022); and (iii) percentage of tree cover around the burrow (Tree), which we hypothesized would positively influence tick occurrence by buffering microclimatic conditions at the entrance of the burrow.

**TABLE 1:** Covariables used to model Florida gopher tortoise burrow *Ornithodoros turicata* infestation probability (*ψ*) and probability of detecting ticks at infested burrows (*p*).

Covariable	Abbreviation	Source	*ψ*	*p*
Topographic Wetness Index	TWI	Estimated using the *wbt_wetness_index* function from whitebox package (Lindsay, 2016). Input 10 m resolution DEM: USGS 1/3 arc‐second Contour (USGS, 2023)	✓	✓
Time since last fire	Fire	OSBS data portal	✓	✓
Percent tree cover	Tree	MOD44B Version 6 Vegetation Continuous Fields for 2021–2022. 250‐m resolution	✓	✓
Burrow temperature	Temp	Measured in the field		✓
Burrow relative humidity	RH	Measured in the field		✓
Burrow size	Size	Width × height. Measured in the field		✓
Sample weight	Sample.W	Measured at time of sample processing		✓
Average soil temperature at study area during the past day	avgTemp_1	FAWN^a^		✓
Average soil temperature at study area during the past 5 days	avgTemp_5	FAWN^a^		✓
Average relative humidity at study area during the past day	avgRH_1	FAWN^a^		✓
Average relative humidity at study area during the past 5 days	avgRH_5	FAWN^a^		✓
Total precipitation at study area during the past day	Prec_1	FAWN^a^		✓
Total precipitation at study area during the past 5 days	Prec_5	FAWN^a^		✓
Sampling occasion	Sampling.occ	NA		✓

^a^ FAWN: Florida Automated Weather Network (https://fawn.ifas.ufl.edu/).

As previous research has shown that relative humidity, temperature and rainfall influence tick activity and movements within the gopher tortoise burrows (Adeyeye & Butler, 1989, 1991), we assessed the influence of weather conditions at and before the time of sampling on the probability of detecting ticks from infested burrows. We used the temperature and relative humidity at the burrow entrance to characterise conditions at the time of sampling. To account for potential delayed responses to weather conditions, we also calculated the average relative humidity, temperature and total precipitation of 24 h and 5 days before the sampling event. As the number of ticks within an infested burrow could also influence the detection probability, we explored variables that could be related to tick abundance. These variables included burrow size, and the three variables described in the paragraph above (Table 1). In addition, we also assessed the influence of the amount of collected substrate (Sample.W) on detection probability. Finally, we explored the possibility of decreased detectability over consecutive sampling events due to population depletion by including the sampling occasion (Sampling_occ, 1 to 3) as a detection covariable.

In fitting the occupancy models, we took a two‐step approach: first we created univariate models of detection probability while holding occupancy probability constant. Then, we kept the variable best accounting for detection probability, while fitting models of covariates for probability of occupancy (MacKenzie et al., 2017). Model selection was based on the Akaike Information Criterion (AIC), taking as similar the models within four AIC units of the lowest AIC. We considered the variables which had a coefficient significantly different from zero at 0.05 probability (MacKenzie et al., 2017). Given that our dataset consisted of 36 sites, we only explored univariate models for both *ψ* and *p*. We fitted the models and evaluated their performance using the functions *occ* and *modSel* in the Unmarked R package (Kellner et al., 2023).

We compared tick detection rates by manual sifting and sieving using a two‐proportion *Z*‐test of samples from infested burrows (i.e., burrows with at least one tick detected in any replicated sampling event). We also compared the number of ticks collected by each of these methods by fitting a Poisson regression of the number of ticks recovered for each positive sample as function of the sampling examination method. For a subset of 22 samples analysed using the sieving protocol, we reported the proportion of ticks collected by each screen size as well as in the bags. We used these data to validate the use of three mesh screens and to determine whether the tray was a required step for collecting all the soft ticks in the sample.

## RESULTS

### Performance of burrow vacuuming and CO_2_ trapping

We completed 92 sampling events across 36 burrows using the vacuum method, and 40 sampling events across 23 burrows using the CO_2_ method. Certain burrows were only vacuumed twice due to the presence of a vertebrate during sampling attempts. *Ornithodoros turicata americanus* was recovered on at least one sampling event at 21 of the 36 burrows for a naïve prevalence (e.g., not accounting for imperfect detection) of 58%. There were 56 sampling events that took place in these 21 infested burrows which resulted in an average proportion of positive replicates per infested burrow of 0.62. The average number of ticks collected from positive sampling events with the vacuum method was 4.3, with a range from 1 to 31. We detected no ticks using the CO_2_ trapping method and therefore discontinued its use for the remainder of the study. No other soft tick species was detected.

### Burrow conditions at time of sampling

The average size of the sampled burrows was 33.82 (SD = 11.41) cm wide by 16.23 (SD = 6.71) cm tall. Burrow depth was not measured as a result of permit restrictions. Although no burrows contained vertebrates at the time of the survey, a gradient of burrow activity was recorded using evidence of tracks, vegetation levels and apron condition. Burrows ranged from no activity, with vegetation and debris covering most or all the apron, to extensive activity, with a clean large apron and vertebrate tracks. The mean relative humidity at the entrance of the burrow at the start of sampling was 56.81% (SD = 9.67%) and the mean temperature was 26.07°C (SD = 3.81°C).

### Tick detection and burrow infestation probability

**FIGURE 3:**
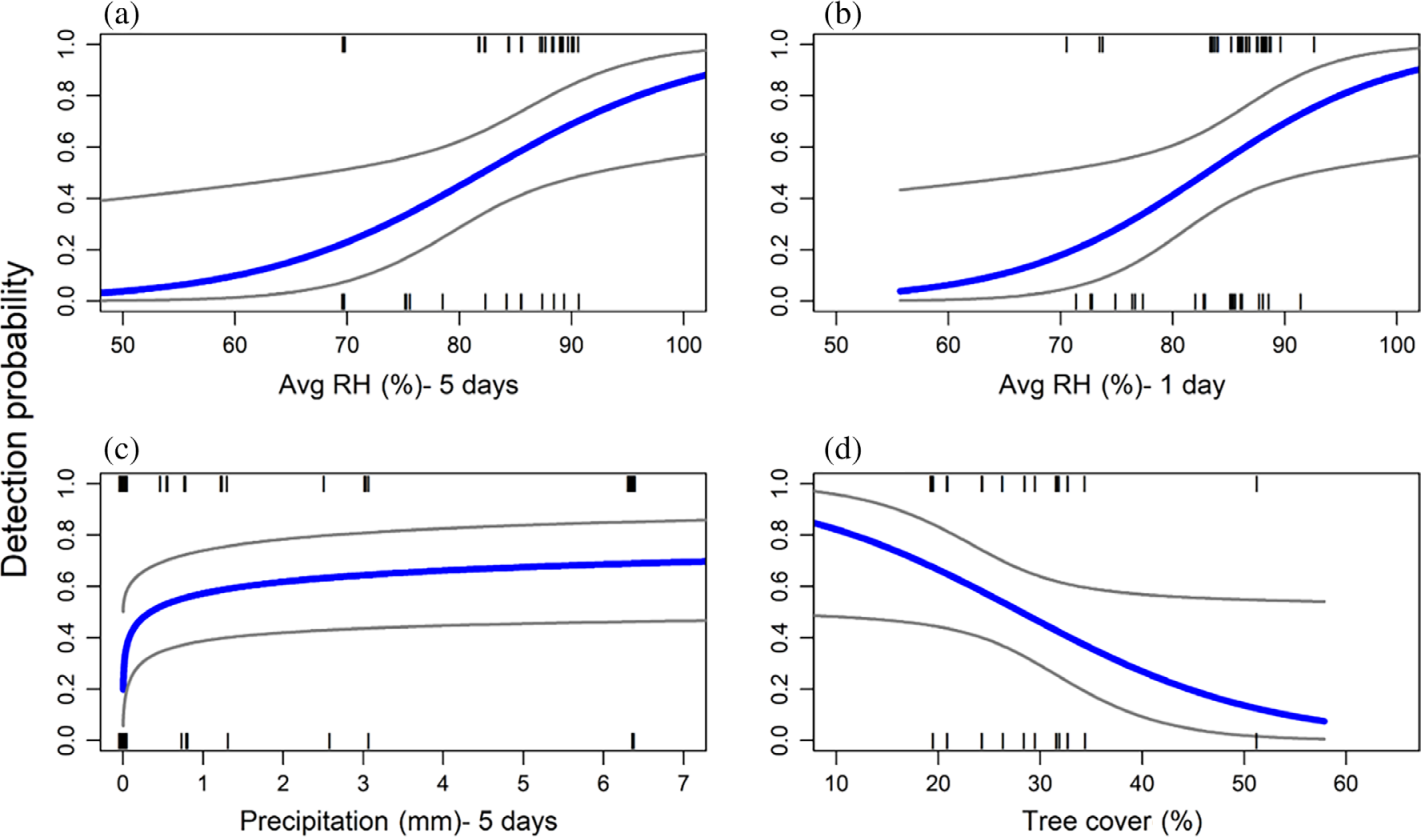
Best supported models describing detection probability of *Ornithodoros turicata* in gopher tortoise burrows in north central Florida. Relationship between detection probability and average relative humidity within 5 days (a) and one (b) day before sampling; total precipitation in the previous 5 days to sampling (c); and percentage of tree cover (d). Grey lines denote the 95% confidence interval and tick marks represent detection/no‐detection at infested burrows.

The best set of models that explained variation in the probability of detection (holding occupancy constant) included relative humidity within the last 5 days, relative humidity within the last day, precipitation within the last 5 days, tree cover and TWI (Table 2). The first three variables showed a positive relationship with detection probability. Tree cover presented a negative relationship, while TWI did not display a significant relationship (Figure 3). The number of previous sampling events (Sampling_occ) was not a strong predictor of the probability of detection (Table 2) suggesting that repeated sampling of the burrow did not deplete the burrow of soft ticks. The best model presented an average detection probability of 53% per sampling occasion. The remaining variables had a similar or higher AIC score as the model with constant detection probability and were not considered further.

**TABLE 2:** Akaike Information Criterion (AIC)‐based model selection of the models exploring detection probability of *Ornithodoros turicata* in Florida gopher tortoise burrows with a constant infestation probability.

Model	nPars	*N*	AIC	Delta	AICwt	cumltvWt
p(avgRH_5)psi(.)	3	36	116.65	0.00	0.29	0.29
p(avgRH_1)psi(.)	3	36	117.34	0.68	0.21	0.50
p(Prec_5)psi(.)	3	36	117.78	1.12	0.17	0.67
p(Tree)psi(.)	3	36	119.07	2.42	0.09	0.76
p(TWI)psi(.)	3	36	120.08	3.42	0.05	0.81
p(Size)psi(.)	3	36	121.09	4.43	0.03	0.84
p(.)psi(.)	2	36	121.16	4.51	0.03	0.87
p(avgTemp_5)psi(.)	3	36	121.43	4.78	0.03	0.90
p(Sampling.occ)psi(.)	3	36	121.94	5.28	0.02	0.92
p(avgTemp_1)psi(.)	3	36	122.46	5.80	0.02	0.94
p(Sample.W)psi(.)	3	36	122.71	6.06	0.01	0.95
p(Temp)psi(.)	3	36	122.87	6.22	0.01	0.96
p(Fire)psi(.)	3	36	122.98	6.33	0.01	0.98
p(Prec_1)psi(.)	3	36	123.11	6.45	0.01	0.99
p(RH)psi(.)	3	36	123.14	6.49	0.01	1.00

*Note*: The variables on weather conditions include averages for the past 5 days (e.g., avgRH_5), past day (e.g., avgRH_1) and at the time of sampling (e.g., RH).

Occupancy models including covariates did not outperform the constant model according to the AIC (Table 3). Although the models including the landscape covariates of occupancy were within two AIC units of the best model, none of these presented a beta coefficient significantly different from zero. Accounting for imperfect detection, the model in which relative humidity within the last 5 days influenced detectability estimated that 70% of the burrows in the study area were infested with *O. turicata*.

**TABLE 3:** Akaike Information Criterion (AIC)‐based model selection of the models exploring *Ornithodoros turicata* infestation of Florida gopher tortoise burrow probability covariables with modelling detection probability as a function of average relative humidity over the past 5 days.

Model	nPars	AIC	Delta	AICwt	cumltvWt
p(avgRH_5)psi(.)	3	116.65	0.00	0.34	0.34
p(avgRH_5)psi(Tree)	4	117.09	0.44	0.27	0.61
p(avgRH_5))psi(TWI)	4	118.08	1.43	0.16	0.77
p(avgRH_5)psi(Fire)	4	118.52	1.86	0.13	0.90

### Sample examination

Using the manual sifting method of tick collection, we examined 24 of the 56 replicates from the infested burrows, of which 14 samples of burrow substrate (58.3%) resulted in tick detection. We collected an average of 6.6 ticks from samples that contained at least one tick. Using the sieving method, we examined the other 32 replicates from infested burrows and detected ticks in 21 of these substrate samples (Table 4; 65.6%). The average number of ticks collected by the sieving method was 2.9 ticks from samples with at least one tick. The probability of detecting at least one tick from infested burrows was not significantly different between the two methods (pval = 0.7). The Poisson regression indicated a significant effect of sample examination method on the number of ticks recovered from infested burrows, with the manual sifting method resulting in a greater number of ticks collected (pval = <0.01). The manual sifting method, however, took longer to complete than the sieving method. For each 3.8 L of substrate, manual sifting took between 45 and 90 min to complete, whereas the sieving system took between 20 and 35 min to complete.

**TABLE 4:** Comparison of two *Ornithodoros turicata* recovery methods from substrate samples collected from Florida gopher tortoise burrows.

	Burrows		Replicates from infested burrows		
	Total	Infested	Total	Positive	Proportion detected (%)
Manual sifting	22	16	24	14	58.3
Sieving	35	20	32	21	55.6
Total	36^a^	21^a^	56^b^	35	‐

*Note*: The first set of columns display the total number of burrows examined by each method and number of these burrows known to be infested. The second set of columns displays the results of examination for samples collected from infested burrows.

^a^ Replicates from a single burrow were examined using different protocols.

^b^ Not all burrows had three replicates completed due to vertebrate presence.

**FIGURE 4:**
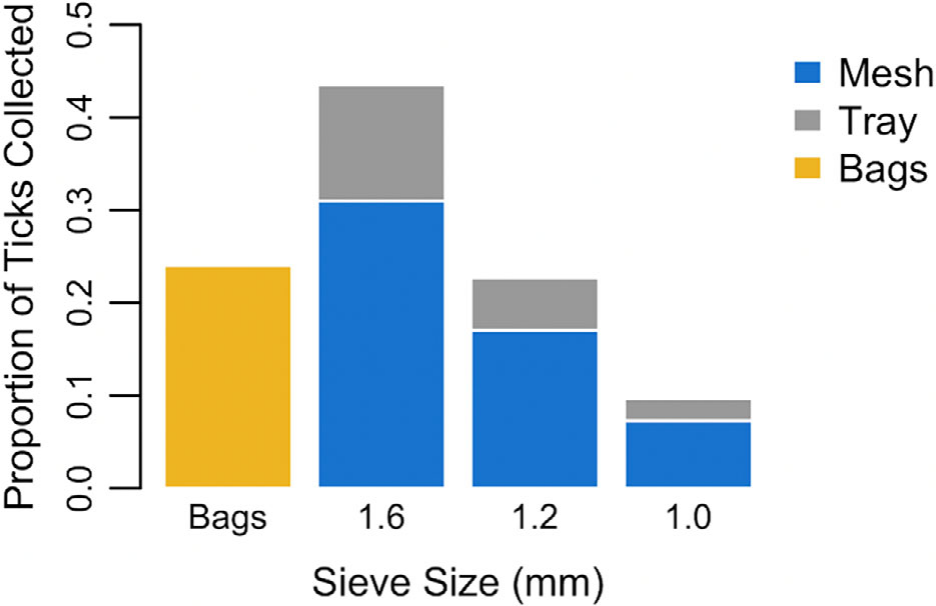
The proportion of the total number of *Ornithodoros turicata* collected using a sieving method (polyethylene plastic bags, Mesh bags, screens), versus tray method (different screen sizes) on gopher tortoise burrow substrate samples.

The sieving method collected ticks in decreasing numbers with each iteration of sampling: 24.1% of ticks were collected during the initial visual examination of the bags; 43.5% of the ticks from the 1.6 ‐mm screen, 22.7% from the 1.2‐mm screen and 9.7% from the 1.0‐mm screen (Figure 4). The examination tray proved to be essential to collecting all the soft ticks that were in each vacuumed sample with 73.2% of the ticks collected from the screens, and 26.8% of the ticks found on the tray that was used after the examination of the screens (Figure 4).

In total, we collected 140 *O. turicata americanus* ticks from OSBS. From this, 15 ticks were identified as larvae, 109 were identified as nymphs and 16 were identified as adults. The adults consisted of 14 females and 2 males.

## DISCUSSION

In this study, we demonstrated that burrow vacuuming is an efficient method of collecting soft ticks and assessing gopher burrow infestation status. For the purposes of rapid and efficient surveillance, this method outperformed the CO_2_ trapping method both in the ability to detect soft ticks and in the time it took to perform the sampling at each burrow. An experienced technician could measure environmental variables and sample a burrow in 10–15 min. By contrast, CO_2_ trapping using our method required 30 min of sampling time for each burrow.

One of the factors that may have impacted the attraction of *O. turicata* to CO_2_ traps was relative humidity. Adeyeye and Butler (1991) found that during the period between June and mid‐November, when mean relative humidity was >60%, soft ticks were more attracted to CO_2_ traps compared to the other half of the year when mean relative humidity was <50%. Their study collected numerous soft ticks with this method throughout the more humid months, while only four ticks were collected in December and none from January through mid‐April. Our study conducted CO_2_ sampling from October to early December and did not collect any soft ticks with this method. However, the mean relative humidity throughout our study period was above 83%, which suggests that there might be additional seasonal variables, or a combination of variables, that could potentially be impacting the attractiveness of CO_2_ or preventing ticks from coming to the surface of the burrow.

Our occupancy framework allowed us to disentangle infestation and detectability and showed that one of the most influential variables on detection probability with the vacuum method was relative humidity. We found that the mean relative humidity from the previous 5 days was positively correlated with the probability of detection of soft ticks in an infested burrow. Five‐day periods with average humidity above 81% resulted in a detection probability of at least 50%. Given the seasonality of soft tick collection success (Adeyeye & Butler, 1991) and the impact of relative humidity for detection probability, time of year and climatic conditions will impact the efficiency of surveillance campaigns and should be considered during the creation of survey designs. Contrary to our expectation, the percentage of tree cover had a negative influence on detection probability, suggesting the need of further work to understand the interplay among tick abundance, host availability and habitat.

Due to permit restrictions, we were only able to vacuum burrow material to a depth of 1 m. This potentially could have impacted our detection results as these ticks are known to move to lower depths during the drier periods of the year. For example, Adeyeye and Butler (1989) found that as much as 70% of ticks collected were 1.5 m and deeper into the burrow during the dry winter months. Nonetheless, burrow vacuuming down to 1 m still achieved our objective of developing a reliable and efficient collection method. Furthermore, there was no evidence of a depletion effect on the detection probability after successive vacuum sampling events, suggesting that this method allows for the exploration of temporal pattern in tick detectability, occurrence and abundance.

The efficiency of the substrate examination methods at detecting an infestation within a burrow were comparable. Both manual sifting and sieving detected an infestation at the same rate; however, the manual sifting method resulted in a higher number of collected individuals. Nonetheless, the sieving was considerably less time‐consuming than the manual sifting method. In many cases, sieving was two to three times faster than the manual sifting method. This trade‐off of saving time at the expense of the number of ticks collected from a burrow can be advantageous when performing large‐scale distribution studies. However, manual sifting may be useful when sampling for pathogen surveillance or tick demographics. The number of samples collected and the time available to examine them should be important considerations when designing a study that requires either of these methods to collect nidicolous ticks.

The use of sieves of different sizes allowed for soft ticks of varying life stages to be collected. Only larval soft ticks smaller than 1.0 mm could fit through the final sieve and avoid collection. As there were no differences in detection rate between sieving and manual sifting, we inferred that omission of the smallest larva did not impact the detection of burrow infestation. Studies that simply assess the presence or absence of soft ticks in burrows might spare the use of the last mesh as it did not impact detectability. Important considerations for the sieving method include the observation that the mesh screen should be examined immediately after sieving while soft ticks are moving towards cover. Once ticks were removed from the mesh, examination of the debris on a white tray proved to be pivotal in collecting large numbers of ticks, accounting for a quarter of the soft tick individuals. Ticks that were inactive or hidden were easily observed on the tray with the UV light.

The combination of burrow vacuuming with sieving was an efficient way of collecting soft ticks from gopher tortoise burrows. Gopher tortoise burrows were targeted as sites of collection based on previous studies and the fact that 66% of the specimens from Florida at the US National Tick Collection are associated with gopher tortoises (Adeyeye & Butler, 1989; USNTC). The use of gopher tortoise burrows allowed for rapid and efficient field collection because the burrows themselves are highly visible and land managers tend to keep updated records of their locations. Nonetheless, we recognise that relying on burrows comes at the cost of missing other possible microhabitats where *O. turicata americanus* could occur. In western populations of this species, infestations have been observed in caves, rock crevices, nests and burrows used by numerous species of different taxa (Kim, 2017). Armadillo (*Dasypus novemcinctus*) and opossum (*Didelphis virginiana*) burrows have been inspected in Florida, but no ticks were recovered from these microhabitats (Adeyeye & Butler, 1989). Regardless, there have not been any systematic studies looking at the infestation of soft ticks in microhabitats other than gopher tortoise burrows in Florida. A microhabitat that should receive special attention in future studies is pigsties where these ticks would be in very close proximity to domestic swine. The vacuuming method could easily be adapted to survey these and other areas, including armadillo and fox dens, cave entrances and other nidicolous locations. The capability of adapting our methodology to different microhabitats including pigsties and other livestock housing makes this standardised protocol useful on a global scale where nidicolous ticks are distributed.

Adeyeye and Butler (1989) reported a high infestation rate of gopher tortoise burrows by *O. turicata* (75%) at a site in central Florida. At our study area in north central Florida, we found similarly high burrow occupancy rates by this tick. Our occupancy model did not find evidence of an effect of topography, fire history or tree cover on the probability of burrow occupancy, suggesting that this tick was ubiquitous on our study area (Estrada‐Peña, 2001; Gleim et al., 2014). If soft ticks are distributed throughout Florida, this ubiquity could result in high potential contact rates with wild pigs which, in turn, could facilitate vector‐borne pathogen transmission. Given the high population densities of wild pigs, the high rate of human assisted movement and the mixing of free ranging wild pigs with pigs in holding facilities (Hernández et al., 2018), the potential role of soft ticks in the maintenance of a disease transmission cycle may be underestimated. Bloodmeal analyses would help clarify the extent that both wild and domestic pigs and soft tick contact occurs.

An optimised method for collecting soft ticks will facilitate large‐scale surveys to better understand the distribution and vectorial capabilities of this species in the United States. Furthermore, the methods presented here could be quickly deployed in large‐scale tick surveys in response to an ASFV outbreak in the southeastern United States. Data provided here on burrow infestation and tick detection probabilities can also be used to design optimal sampling schemes for regional scale occupancy and distribution models of soft ticks (Bailey et al., 2007). Bloodmeal analyses of collected ticks would further elucidate tick–host relationships and the potential role soft ticks could play in the transmission dynamics of ASFV. Pathogen surveillance for other pathogens, such as *Borrelia turicatae* and *Leptospira* sp., could also be conducted (Burgdorfer, 1956; Krishnavajhala et al., 2018). This standardised and rapid survey method constitutes an important tool to prepare for and rapidly respond to the potential introduction of ASFV in the southeastern United States and could be readily adapted for surveillance in any region of the United States.

## AUTHOR CONTRIBUTIONS

**Nicholas Canino:** Writing – original draft; investigation; methodology; formal analysis; visualization; conceptualization. **Carson Torhorst:** Conceptualization; writing – review and editing; methodology; investigation; supervision; validation. **Sebastian Botero‐Cañola:** Conceptualization; data curation; formal analysis; visualization; writing – review and editing; supervision; investigation; methodology; validation; writing – original draft. **Lorenza Beati:** Methodology; writing – review and editing; resources; validation. **Kathleen C. O'Hara:** Conceptualization; writing – review and editing; project administration; funding acquisition. **Angela James:** Conceptualization; project administration; writing – review and editing; funding acquisition. **Samantha M. Wisely:** Conceptualization; writing – review and editing; project administration; supervision; funding acquisition; resources.

## FUNDING INFORMATION

This research was funded by United States Department of Agriculture, Animal and Plant Health Inspection Service, Center for Epidemiology and Animal Health award identification number AP22VSSP0000C050. Additional funding was provided by the USDA National Institute of Food and Agriculture, McIntire‐Stennis project 7004318. The findings and conclusions reported are those of the author(s) and should not be construed to represent any official USDA or U.S. Government determination or policy.

## CONFLICT OF INTEREST STATEMENT

The authors declare no conflicts of interest.

## Data Availability

The data that support the findings of this study are available on request from the corresponding author. The data are not publicly available due to privacy or ethical restrictions.
